# Penile Mondor’s disease

**DOI:** 10.1186/2051-4190-24-5

**Published:** 2014-03-03

**Authors:** Hakan Öztürk

**Affiliations:** Department of Urology, School of Medicine, Sifa University, Fevzipasa Boulevard No:172/2 Basmane, Konak, 35240 Izmir, Turkey

**Keywords:** Penile mondor’s disease, Causes, Diagnoses, Treatments, Maladie de Mondor pénienne, Ĕtiologies, Diagnostics, Traitements

## Abstract

Mondor’s disease is a rare, self-limiting, benign process with acute presentation characterized by subcutaneous bands in several parts of the body. Penile Mondor’s disease (PMD) is thrombophlebitis of the superficial dorsal vein of the penis. It is usually considered as thrombophlebitis or phlebitis of subcutaneous vessels. Some findings suggest that it might be of lymphatic origin. The chest, abdominal wall, penis, upper arm, and other parts of the body may also be involved by the disease. Although its physiopathology is not exactly known, transection of the vessel during surgery or any type of trauma such as external compression may trigger its possible development. This disease almost always limits itself. It may be associated with psychological distress and sexual incompatibility. The patients usually feel the superficial vein of the penis like a hard rope and present with complaint of pain around this hardness. Diagnosis is usually easy with physical examination but color Doppler ultrasound examination is important for differential diagnosis. Thus, a close collaboration is required between radiologist and urologist in order to determine the correct diagnosis and appropriate therapies.

## Résumé

La maladie de Mondor est une entité rare, auto-limitée et bénigne, de présentation aiguë, qui est caractérisée par la présence de cordons sous-cutanés en différentes régions du corps. La maladie de Mondor pénienne est une thrombophlébite de la veine dorsale superficielle du pénis. Elle est habituellement considérée comme une thrombophlébite ou une phlébite des vaisseaux sous-cutanés. Quelques éléments suggèrent qu’elle pourrait être d’origine lymphatique. La paroi latérothoracique, la paroi abdominale, le pénis, le bras et d’autres parties du corps peuvent être touchés par la maladie. Cette maladie est presque toujours auto-limitée. Bien que la physiopathologie n’en soit pas précisément connue, la section transversale d’un vaisseau lors d’une chirurgie ou n’importe quel type de traumatisme, telle qu’une compression externe, peuvent constituer le déclencheur d’un possible développement. Elle peut être associée à une détresse psychologique et des difficultés sexuelles. Le patient ressent habituellement cette veine superficielle du pénis comme un cordon dur et se plaint d’une douleur autour de cette induration. Le diagnostic en est habituellement facile à l’examen clinique, mais l’examen par écho-doppler couleur est important pour le diagnostic différentiel. Aussi, une étroite collaboration est requise entre le radiologiste et l’urologue pour poser un diagnostic correct et mettre en place les traitements appropriés.

## Materials and methods

Literature search was made through a search in the MedLine database using PubMed and Scopus for the articles which were written in English language and published between July 1988 and June 2013. Electronic search was limited to the following keywords: *penile mondor’s disease (penile superficial venous thrombosis), causes, symptoms, diagnosis and treatment.* A total of 49 articles were found about Penile Mondor’s disease of which 34 were specific to it. Six of them were compiled articles. No meta-analysis was found. The last compilation was three years ago.

## Results

### Definition and epidemiology

Mondor’s disease is thrombophlebitis of the superficial veins and was first defined by Henri Mondor in the superficial veins of the chest wall in 1939 [1]. In 1955, Braun-Falco defined dorsal phlebitis of the penis in the context of generalized phlebitis [2]. Isolated thrombosis of the dorsal superficial vein of the penis was first reported and Penile Mondor’s disease was defined by Helm and Hodge in 1958 [3]. Penile Mondor’s disease is a rare and under-recognized benign genital condition. Its real incidence is considered to be higher than reported. Only 53 cases were reported in the literature [4]. Penile Mondor’s disease (PMD) affects sexually active men of any age. Age of the patients range between 18 and 70 m [4]. No specific etiology has been found in the cases reported so far.

### Etiology and pathogenesis

Penile venous system; Venous drainage of the penis begins at the base of the glans; a series of venous canals merge to form the dorsal vein of the penis, which in turn runs along a groove between the corpora and drains into the preprosthatic venous plexus. The circumflex veins orginate in the corpus spongiosa and extend around the corpus cavernosum on either side to merge with the deep dorsal vein perpendicularly. They are only present in the two distal thirds of the penis and total between 3 and 10 in number. Intermediate venules of the venous sinuses in turn form and drain into a capillary plexus beneath the tunica. This plexus system gives rise to emitting veins that generally extend obliquely between the layers of the tunica and drain into the circumflex vein at the dorsolateral level. The emitting veins in the proximal third of the penis merge over the dorsomedial surface of the corpus cavernosum bilaterally to form between 2 to 5 cavernous veins. In the hilum of the penis, these vessels pass between the pillars and the bulbar region receiving branches from each of them and joining with the internal pudendal veins [5]. Apart from the erectile structures, vessels of the penile skin also originate from the external pudendal branch of the femoral artery. These vessels enter the penis at radix of penis. They run longitudinally in the Dartos layer and make frequent anastomoses between each other [6]. This widespread venous network may be affected by trauma and inflammatory or infectious processes in the genital region under certain conditions. Causes of the disease include frequent, severe, and prolonged sexual intercourse, penile trauma, prolonged sexual abstinence, local (e.g. syphilis, candida infections) or distant infections, history of sexually transmitted diseases, thrombophilia, repair of inguinal hernia, orchiopexy, varicoselectomy, use of intracavernous drugs, use of vacuum, Behçet’s disease, body building exercises, cancer in the pelvic region, metastatic pancreas cancer and migratory phlebitides due to paraneoplastic syndromes, venous occlusion caused by filled bladder, abuse of intravenous drugs, and tendency to thrombosis [7, 8]. (Table 1) Many predisposing factors may lead to thrombosis of dorsal vein of the penis. Thrombosis occur as a consequence of intravascular coagulation due to injury to vessel wall, stasis, and hypercoagulation known as Virchow’s triad [7]. But the main etiological cause is considered to be trauma due to sexual intercourse [9].

**Table 1:** **Causes of penile Mondor’s disease**

Traumatic etiology	Infectious etiology	Surgical etiology	Oncologic etiology	Other
**• Causes of the disease include frequent, severe, and prolonged sexual intercourse**	**• Syphilis**	**• Repair of inguinal hernia**	**• Cancer in the pelvic region**	**• Use of intracavernous drugs**
**• Penile trauma**	**• Candida infections**	**• Orchiopexy**	**• Metastatic pancreas cancer and migratory phlebitides due to paraneoplastic syndromes**	**• Abuse of intravenous drugs**
**• Prolonged sexual abstinence**	**• Distant infections**	**• Varicoselectomy**		**• Tendency to thrombosis**
**• Use of vacuum**	**• History of sexually transmitted diseases**			**• Thrombophilia**
**• Body building exercises**	**• Behçet’s disease**			**• Venous occlusion caused by filled bladder**

### Symptoms

Symptoms caused by PMD don’t show distinctive features. There are asymptomatic cases. The patients usually present with an hardness like a rope at dorsum of the penis. They complain of episodic or continuous pain and throbbing. Erythema and edema may be seen on the penile skin. Some patients feel distention on the site of thrombosis. There is usually pain typically exacerbated during erection. Some patients may develop irritative voiding symptoms [10, 11].

### Diagnosis

Penil Mondor’s disease may be diagnosed with medical history and physical examination. Color Doppler ultrasound examination is of importance in differential diagnosis. Patients diagnosed using Magnetic Resonance Imaging (MRI) have also been reported [12]. Histopathological examination may also be helpful in diagnosis [1]. Penil Mondor’s disease may be divided in three separate clinical stages as acute, subacute, and re-canalized. The acute form tend to develop in men between 20 and 40 years old typically in 24 hours following prolonged sexual intercourse. It probably develops secondarily to vascular endothelial trauma.

### History and physical examination

**Figure 1:**
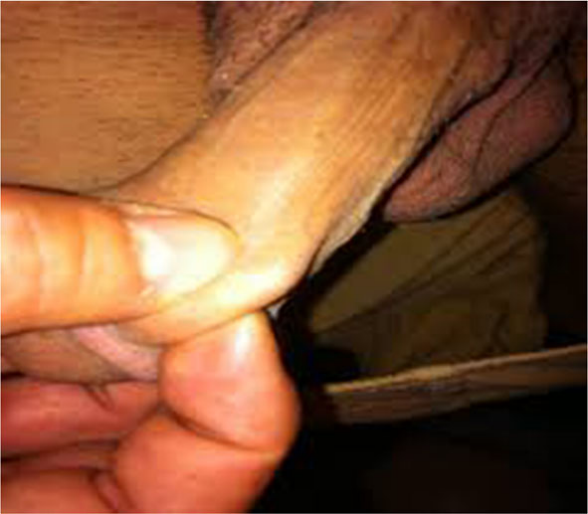
**The palpable thrombotic superficial vein of the penis.**

The patients present with a palpable lesion as a thick cord occurring 24 to 48 hours after a prolonged sexual intercourse (Figure 1). Some of these patients have history of one or more sexually transmitted diseases. The lesion is on the dorsum of penis in all of the patients. It is close to radix of the penis in many patients while it is close to sulcus coronarius in some patients [13]. Length of the thrombotic vessel may be 2 to 10 cm. The lesion usually extends to the suprapubic region. The thrombosed vessel is adhered to the penile skin covering it. The vessel may appear as swollen and erythematous. The thrombotic vessel may occasionally be seen in the superficial pudendal vein. This condition is considered as a variant of Mondor’s disease [14].

### Laboratory investigations

There are no typical changes in the serum. Laboratory investigations showing tendency to thrombosis are rarely required. Systemic tests such as Anti-thrombin III, deficiency of Protein C and Protein S, MTFR gen defect, and assays for thrombophilia are more valuable in the cases of widespread thrombophlebitis.

### Pathology

In pathological examination, the most important differential diagnosis is sclerosing lymphangitis of the penis. It is very easy for the microvascular structures to be confused with the lymphatic system. Immunohistochemical investigations may separate them. Histopathological examination may reveal swelling in the endothelial cells, increased connective tissue in the vessel wall, thrombus, and infiltration of the lymphocytes, histiocytes, and the plasma cells in the perivascular area. Differential diagnosis with sclerosing lymphangitis is made by staining of the endothelium with monoclonal antibodies to CD31 and CD34. No staining with these antibodies occur in sclerosing lymphangitis.

### Color Doppler ultrasound examination

Color Doppler ultrasound examination plays important role in differential diagnosis of two conditions of the penis: Mondor’s disease and non-venereal sclerosing lymphangitis of the penis. In Doppler Ultrasound (US) examination, dorsal vein thrombosis and hemodynamic changes due to it are observed. Although findings of color Doppler US are considered to suffice for diagnosis of Mondor’s disease with appearance of superficial dorsal vein thrombosis without flow signals in this region, some studies have suggested that this is not sufficient for diagnosis. In addition to classical findings reported previously, cavernous arterial flow-signal pattern has currently been defined for Mondor’s disease. On the color Doppler US, no color filling and flow spectrum is observed in the lumen of superficial vein. Color Doppler US examination of the cavernous arteries reveal low-speed, high-resistance flow pattern. These color Doppler findings aren’t observed in sclerosing lymphangitis [15].

### Pelvic MRI

Cases diagnosed with magnetic resonance angiography have been reported in the literature [12]. But it is not used routinely in diagnosis and differential diagnosis of Mondor’s disease. History and physical examination is of primary importance in the diagnosis. Color Doppler US may be used when necessary. But role of the MRI is controversial.

### Differential diagnosis

The most important conditions in the differential diagnosis are Peyronie’s disease and sclerosing lymphangitis. Both physical examination and color Doppler US reveal that the hardness is not on the tunica albuginea. Sclerosing lymphangitis is a more common condition. Color filling and flow-spectrum are not observed in the lumen in the color and spectral examination of the penile dorsal vein. Low-speed, high-resistance flow pattern is observed in the cavernous arteries. This finding of color Doppler US is absent in sclerosing lymphangitis. Furthermore, a folded and irregular hardness is found in sclerosing lymphangitis whereas the thrombosis is palpated as a smooth spermatic cord in Mondor’s disease.

### Treatment

Penile Mondor’s disease is clinically divided in three stages as acute, subacute-chronic and recanalization stages [16–18]. In many cases, it recovers within 4 to 6 weeks with the vessel re-gain permeability in 9 weeks without any medical treatment.

### Medical treatment

In the acute stage, sexual activity should be restricted in addition to the use of anti-coagulant agents. Creams containing heparin and anti-inflammatory drugs are used in the subacute and chronic stages. During these stages, the patient should be advised to restrict his sexual activity until the symptoms due to infection if exists, and severe pain disappear. No permanent sequel has been defined in long-term.

### Surgical treatment

Thrombectomy and resection of the superficial penile vein are applied surgically in the patients refractory to the medical treatment. Symptomatic patients without flow in the color Doppler US after the sixth week should be considered as refractory to the medical treatment. Option of surgical treatment should be offered.

In conclusion, Penil Mondor’s Disease is a rare clinical condition that every urologist should know. Early and accurate diagnosis increases efficiency of the medical treatment and limits necessity of the surgical options. In this respect, a well performed physical examination and knowing findings of color Doppler US exam is of great importance in early diagnosis and treatment. Problems such as anxiety and sexual dysfunction may occur in the patients with penil Mondor’s disease. Thus accurate and rapid diagnosis and treatment is of great importance.

## References

[1] Niechajev I (2013). Mondor’s subcutaneous banding after transaxillary breast augmentation: case report and the review of literature. Aesthetic Plast Surg.

[2] Bird V, Krasnokutsky S, Zhou HS, Jarrahy R, Khan SA (1997). Traumatic thrombophlebitis of the superficial dorsal vein of the penis: an occupational hazard. Am J Emerg Med.

[3] Helm JD, Hodge IG (1958). Thrombophlebitis of a dorsal vein of the penis: report of a case treated by phenylbutazone (Butazolidin). J Urol.

[4] Hamilton J, Mossanen M, Strote J (2013). Mondor’s Disease of the penis. West J Emerg Med.

[5] Rodriguez-Faba O, Parra Muntaner L, Gomez Cisneros SC, Martin-Benito JL, Escaf-Barmadah S (2006). Thrombosis of the dorsal vein of the penis (Mondor’s phlebitis). A case report. Actas Urol Esp.

[6] Benson GS, Boileau M, Gillenwater JJ, Grayhack JT, Howards SS, Mitchell ME (2002). The penis: sexual function and dysfunction. Adult and Pediatric Urology.

[7] Griger DT, TE A o, Grisier DB (2001). Penile Mondor’s disease in a 22-year-old man. J Am Osteopath Assoc.

[8] Zor M, Tahmaz L, Basal S, Irkılata HC, Dayanc M (2009). Penile Mondor’s disease in a 32-year-old man. Turkiye Klinikleri J Med Sci.

[9] Kumar B (2013). Penile Mondor’s disease: an underdiagnosed and under-reported benign condition. Sex Transm Infect.

[10] Sasso F, Gulino G, Basar M, Carbone A, Torricelli P, Alcini E (1996). Penile Mondors disease: an underestimated pathology. Br J Urol.

[11] Thomazeau H, Alno L, Lobel B (1983). Thrombosis of the dorsal vein of the penis. A propos of two cases. J Urol.

[12] Boscolo-Berto R, Iafrate M, Casarrubea G, Ficarra V (2009). Magnetic resonance angiography findings of penile Mondor’s disease. J Magn Reson Imaging.

[13] Al-Mwalad M, Loertzer H, Wicht A, Fornara P (2006). Subcutaneous penile vein thrombosis (Penile Mondor’s Disease): pathogenesis, diagnosis, and therapy. Urology.

[14] Conkbayir I, Yanik B, Keyik B, Hekimoğlu B (2010). Superficial dorsal penile vein thrombosis (Mondor disease of the penis) involving the superficial external pudendal vein: color Doppler sonographic findings. J Ultrasound Med.

[15] Han HY, Chung DJ, Kim KW, Hwang CM (2008). Pulsed and Color Doppler sonographic findings of penile Mondor’s disease. Korean J Radiol.

[16] Swierzewski SJ, Denil J, Ohl DA (1993). The management of penile Mondor’s phlebitis: Superficial dorsal penile vein thrombosis. J Urol.

[17] Tanii T, Hamada T, Asai Y, Yorifuji T (1984). Mondor’s Phlebitis of the penis: a study with factor VIII related antigen. Acta Derm Venereol.

[18] Özkara H, Akkuş E, Alıcı B, Akpınar H, Hattat H (1996). Superficial dorsal penile vein thrombosis (Penile Mondor’s Disease). Int Urol & Nephr.

